# XRMA analysis and X-ray diffraction analysis of dental enamel from human permanent teeth exposed to hydrogen peroxide of varying pH

**DOI:** 10.4317/jced.55618

**Published:** 2019-06-01

**Authors:** Nina Sabel, Andreas Karlsson, Lennart Sjölin

**Affiliations:** 1 Department of Pediatric Dentistry, Institute of Odontology, Sahlgrenska Academy, University of Gothenburg, Gothenburg, Sweden; 2 Department of Medical Epidemiology and Biostatistics, Karolinska Institutet, Stockholm, Sweden; 3 Department of Chemical and Biological Engineering, Chalmers University of Technology, Göteborg, Sweden

## Abstract

**Background:**

This *in vitro*investigation shows how 3.3% H2O2, at different pH-values affects the enamel.

**Material and Methods:**

A number of fifteen human premolars were used. The enamel of the coronal half in six of the teeth, were exposed by H2O2. Nine teeth were prepared to enamel powder. The enamel was exposed to 3.3% H2O2, at six different pH-values (pH range 4.5 - 7.0). Analyses of the topography of enamel performed by scanning electron microscope (SEM) and the chemical composition of enamel by X-ray microanalysis (XRMA). X-ray powder diffraction (XRD) analysed the crystallinity in enamel powder.

**Results:**

The exposure to H2O2 at pH<5.5 resulted in a rougher topography of the enamel, according to the SEM studies. The XRMA analysis revealed a increase in the ratio of Ca:C. Exposure to H2O2 at pH>5.5 resulted in a decrease of O in the exposed enamel, and changes in C:P, Ca:C, Ca:P and Ca:O were observed. The H2O22 did not affect the unit cell parameters, but the signal-to-noise level was increased for slightly acidic or neutral solutions. The unit cell parameters decreased in the acidic solutions.

**Conclusions:**

The exposure to H2O2 at varying pH values affect the enamel with two different mechanisms. One effect is the oxidation of the organic or bioorganic matter in the hydroxyapatite matrix, due to the use of 3.3% H2O2. The other effect is due to the current pH of the H2O2, since the structure of the hydroxyapatite starts to erode when the pH<5.5.

** Key words:**Dental Enamel, Tooth Bleaching Agents, Hydrogen Peroxide, Scanning Electron Microscopy, X-ray diffraction.

## Introduction

The mechanism of the hydrogen peroxide is driven by oxidation of the organic or bioorganic substances in enamel and in dentine (1). This chemical oxidation contributes to the largest increase of lightness of the tooth, compared to other methods, e.g., deproteinization and/or the subsequent demineralization (2). The most accepted theory behind the whitening of teeth is due to the low molecular weight of the reactive oxygen radical (O22-); the hydrogen peroxide contributing to a quick penetration through the enamel, reaching the dentin soon after application, and eventually the pulp (3-6). The reaction of this radical, with chromophores or particles of pigmentation (organic and bioorganic matter) in the enamel and dentin, results in smaller residual products from the oxidation process that resolve from the environment of the teeth, and provide the person with the appearance of whiter teeth.

During treatment, the surface of the enamel is in immediate contact with the peroxide. In previous investigations, the topography of the enamel has been evaluated for changes, when exposed to peroxide (7-10). The peroxide influences the topography of the enamel surface irrespective of the pH-value and results in micro-morphological changes of the enamel surface (7-9).

An important factor of the whitening therapy is the pH-value. The chemical erosion of enamel normally occurs due to an attack of a proton (H+) from a weak or strong acid. Erosion might occur when a complex anion binds to the calcium ion in the hydroxylapatite matrix, thus locally destroying the structure (11).

Several previous studies focused on observing the effects of whitening products on the enamel surface, considering the concentration of hydrogen peroxide rather than the pH-value (12). As previously stated, the pH of whitening agents is of great importance for the effects on enamel, e.g., in a previous study, the topography of enamel revealed a severe alteration when exposed to carbamide peroxide at a low pH, compared to neutral pH (13). An elegant summary of the hydroxylapatite saturation as a function of pH, including the concentration parameters of other available ions, has been conducted by Lussi *et al.*, showing the schematic dissolution of enamel (11), however, the combination of pH and hydrogen peroxide concentrations is lacking in the study.

The effect of whitening products on the structure of enamel has not been thoroughly investigated or determined, when focusing on how enamel is affected by moderately concentrated peroxide in combination with different pH- values. Subsequently, the aim of this study was to investigate the importance of the pH-value when hydrogen peroxide is utilized in the whitening treatment. The topography of the enamel, using SEM methods parallel with the analysis of the structure, as well as the crystallinity in enamel exposed to hydrogen peroxide using XRD, has not yet been studied to the authors’ knowledge.

This study is directed toward the analysis of the enamel including the topography of the surface, the chemical content and analysis of the crystallinity, and the structural composition of the enamel, when exposed to hydrogen peroxide at different pH-values (ranging from 4.5 - 7.0). In addition, the null-hypothesis used in this study is that the oxidation effect of hydrogen peroxide on enamel, at different pH-values, will be determined independently from the pH-value of the exposing agent.

## Material and Methods

-Tooth samples

Fifteen premolars, extracted from patients undergoing orthodontic treatment, were collected. The teeth were stored in physiological NaCl solution until the start of further preparation. After polishing the enamel thoroughly with pumice and water, the teeth were rinsed in de-ionized water for five minutes and left to dry, in air in room temperature, for one hour.

In six of the premolars, a hole was drilled through the root in order to create a hanging arrangement. A mark was made at half the crown height with a low-speed diamond drill and a permanent marker.

Nine teeth were used for the preparation of enamel powder. The enamel was ground off using a low-speed dental handpiece and a diamond bur. Enamel powder from the nine premolar teeth was pooled into one batch and thereafter divided into seven samples (≈0.2g each) in Eppendorf tubes, marked A-G.

A solution of 3.3% H2O2 was adjusted with 3M NaOH and HCl, to six different pH levels from 4.5 to 7.0, in approximately 0.5 pH unit intervals. The pH-value was measured with a pH-meter (Accumet®, Fischer Scientific, Pittsburg, USA) at room temperature. The value of the critical pH for the dissolution of enamel was preliminarily set between pH ≤ 5.5 and pH ≥ 6 in this study, while no calcium or phosphate ions were present in the reservoir solutions.

Six small glass flasks were used for the experimental procedures, numbered 1-6. After filling the flasks with 2 ml of 3.3% H2O2 of the respective pH-values, each tooth was hung up with dental floss with the cuspal part downward, thus exposing the cuspal half of the crown to the solution, up to the predefined mark. The cervical part of the crown was still unexposed and used as a reference for each tooth. After securing the dental floss, the teeth were exposed to the solution for 24 hours in room temperature.

After exposure to hydrogen peroxide, the teeth were washed with de-ionized water in an ultra-sonic bath for five minutes and then left to dry, in air. Photos of the teeth were taken in a Leica M80 stereo microscope (Leica Microsystems, Wetzlar, Germany) at low magnification (x0.75) in incident light, using a Leica digital camera (Leica DFC420 C, Leica Microsystems, Wetzlar, Germany) with the Leica Application Suite LAS V3.7.0 (Leica Microsystems, Heerbrugg, Switzerland). The photos were used for orientation of the samples in the scanning electron microscope (SEM).

The powder, in tubes marked A-F, was exposed to 3g of 3.3% H2O2 (solution with pH ranging 4.5-7.0) for one minute. The last enamel sample (tube G) was unexposed and used as reference for all experiments.

After the enamel powder had been exposed to H2O2, 30ml de-ionized water was added to the mixture, which then was filtered using a glass filter. The enamel powder was first treated with 95% ethanol and then left to dry in regular air at room temperature for five minutes, in order for the water to evaporate.

The analysis of the enamel powder was carried out in an X-ray diffraction instrument (XRD) for identification of the crystal mineral phases in the six exposed enamel samples (A-F), as well as in the reference enamel powder samples (G,H).

The teeth were oriented in two-part polypropylene mounting cups for resins (FixiForm, Struers ApS, Ballerup, Denmark), using a fast-curing clear acrylic cold mounting resin (ClaroCit®, Electron Microscopy Sciences, Fort Washington, PA, USA), which also prevented vaporization of resin onto the enamel surface. Finally, the teeth mounted in the acrylate were turned upside down and rearranged in the mounting cups. The remaining volume of the mounting cups were filled with an epoxy resin (Epofix®, Electron Microscopy Sciences, Fort Washington, PA, USA), in order to create a socket for the microscope.

-Scanning electron microscopy (SEM) and X-ray micro analysis (XRMA)

The samples were coated with gold in a plasma coater with a thickness of ≈25nm. The XRMA analysis was performed in a Hitachi VP-SEM S-3400N (Hitachi, Tokyo, Japan), equipped with an Oxford EDS system and INCA Energy software (Oxford Instruments, Abingdon, UK.). All analyses were carried out at 20 kV accelerating voltage and the working distance from sample to electron optical column was 9.6 mm, with a tolerance of ±0.1 mm. The beam was aligned in the “Microscope setup” using “Wave” in INCA when the microscope was positioned in a Faraday cage in the specimen holder. The beam current was adjusted to 6.0 nA ±0.1nA and checked every two hours.

The XRMA analysis was carried out as previously described by Melin *et al.* (14).

Images were taken in rectangular areas in magnifications up to 2000x from four different areas in the exposed and non-exposed enamel from each tooth sample. For the elemental analysis of C, O, Na, Mg, P, Cl, K and Ca, the “All elements” and “Normalized” options in the INCA software were used and a spectrum was acquired with a live time of 100 seconds. The measurements were carried out in a magnification of 1000x. In each sample, measurements were made in rectangular areas (85x125 µm); four in the exposed and four in the unexposed enamel, respectively. Thereby, each tooth provided its own reference. Normalized values of the elements were calculated by the INCA software, and all measurements were considered to be semi-quantitative.

-X-ray diffraction analysis (XRD)

The X-ray diffraction analyses were performed in a powder diffractometer D5000, produced by Siemens AXS, with CuKα radiation (45kV, 40mA, λ=1.5407Å) in Bragg-Brentano geometry, using a variable divergence slit at the primary side, which allowed illumination of the sample surface in a circle with a diameter of 20mm and curved secondary monochromator, and a scintillation detector. The measurement was made using the coupled scan in region 20-60o in 2Ɵ, using step size 0.05o, with a ten-second measurement in time/step.

The powder was placed in the diffractometer and the related diffractograms were collected. After an inspection and critical examination of each diffractogram, the peaks were indexed, based on a least squares refinement fit of the data. Selected peaks were distinguished and their corresponding 2Ɵ and d- values were subsequently calculated.

-Statistics

Statistics were calculated by IBM® SPSS® Statistics, version 22, IBM Corporation 1989 & 2013. The paired sample t-test was used when the comparison was made in the same tooth sample. Independent samples t-test calculations were utilized when comparisons between different tooth samples were undertaken, then setting the p-value according to *p*<0.05.

-Ethical considerations

The premolars used in the study are from healthy individuals, which were planned to undergo orthodontic treatment with fixed appliances. Teeth were donated by free will and were stored under humid conditions in a refrigerator, without reference to any specific patient.

## Results

-SEM images

**Figure 1:**
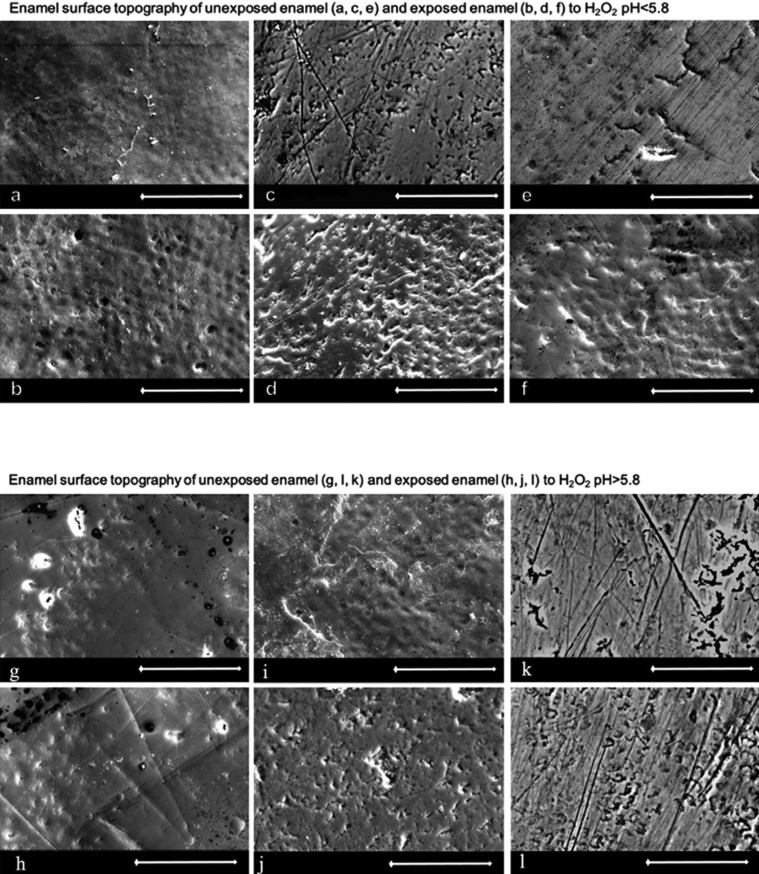
SEM images (magnification 1000x; white bar = 50 µm) of the enamel surface topography. Images of unexposed enamel (a, c, e) and exposed enamel (b, d, f) to H2O2 pH<5.5. The relief of the enamel prisms are clearly discernable in the exposed enamel (b, d, f). The pitted appearance is particularly pronounced in enamel exposed to H2O2 at acidic pH values. Images of the enamel surface topography of unexposed enamel (g, i, k) and exposed enamel (h, j, l) to H2O2 pH>5.8. The exposed enamel (h, j, l) shows a surface of enamel without debris of organic material, where scratches and marks of the surface are detectable.

The SEM images were taken from the exposed and unexposed surfaces of each tooth at 1000x (Fig. 1a). The images of the topography reveal irregularities on the surface pattern. Compared to the unexposed areas, the exposed areas appear to have a rougher surface structure. For the enamel samples exposed to H2O2 with pH<5.5, the relief of the prism structure seems to be more distinct. The pattern of the prism structures is visualized as a pattern of concave pits, constituting the entire exposed area to a varying, uneven extent. Notably, the pits are numerous and tightly arranged.

The SEM images show a surface of irregularities and scratches in enamel exposed to H2O2 at pH>5.5 (Fig. 1b). The prism pattern is detectable in the non-exposed and exposed enamel to an equal extent. It is indicated that the surfaces of the exposed enamel unmask scratches, compared to the unexposed enamel.

The prism at the enamel surface, visible as tightly arranged pits, is more pronounced in the exposed teeth samples being treated with H2O2 at pH<5.5, compared to the teeth exposed to H2O2 with pH>5.5.

-XRMA measurements

The results from the XRMA analyses, for each of the six pH-values, are calculated as mean values for the measured elements from the four areas of unexposed and exposed enamel. In addition, the results featured from the calculation are the mean values from the pooled groups of teeth exposed to H2O2 with pH<5.5 and to pH>5.5, respectively ( Table 1).

**Table 1:**
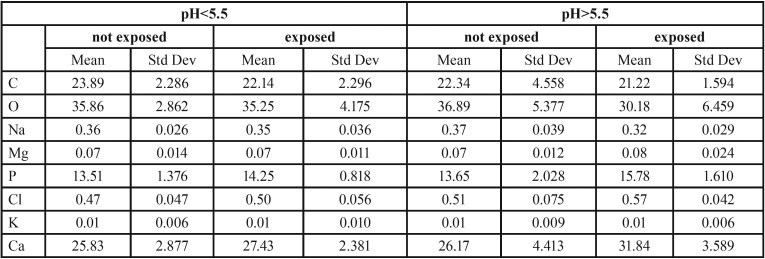
Semiquantitative values of elements in unexposed enamel and when exposed to H2O2 pH<5.5 or H2O2 pH>5.5.

The semi-quantitative values for the elements C, O, Na, Mg, P, Cl, K and Ca of exposed enamel, in the pooled groups, were compared to the same elements from the unexposed enamel in the group. The mean values from the semi-quantitative analysis are presented in  Table 1. When the chemical content of unexposed and exposed enamel in the pooled group at pH<5.5 was analyzed and compared, no significant differences were detected.

A difference in the chemical content was found when the pooled group of H2O2 at pH>5.5 was compared with unexposed enamel. There was a significant difference in the content of O, Na, P, Cl and Ca when using a paired sample t-test with *p*<0.05 ( Table 1).

**Figure 2:**
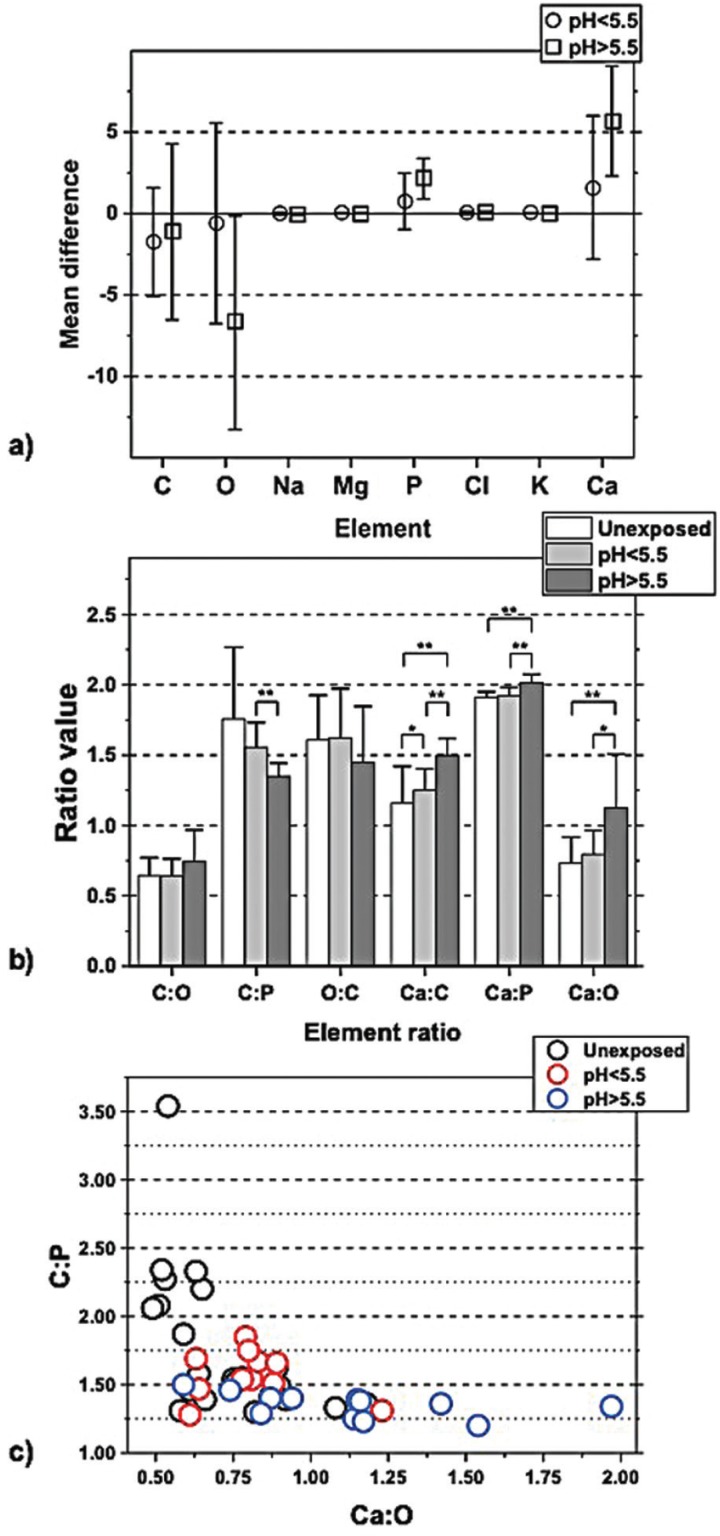
a. Difference calculated for each element between exposed and unexposed enamel; the pooled pH-groups are shown separately. Higher content of O was seen in enamel exposed to H2O2 pH>5.5 (*p*<0.05 paired sample t-test), compared with the unexposed enamel. Additionally, a lower content of P and Ca was seen in the enamel exposed to H2O2 pH>5.5, *p*<0.05 paired sample t-test. b. Elemental ratios of unexposed enamel and exposed enamel of the two pooled pH-groups. Comparing the ratios in the exposed enamel of the two pooled pH-groups with independent sample t-test showed difference in ratios of C:P, Ca:P, Ca:O, and Ca:C. The ratio of C:P is lower in pH>5.5, *p*<0.01. Higher ratios were found of Ca:C, *p*<0.05 in enamel pH<5.5 compared with unexposed enamel.Higher ratios were found of Ca:P and Ca:C, *p*<0.001 and of Ca:O, *p*<0.05 in enamel pH>5.5 compared with unexposed enamel. c. Values for ratio C:P plotted in a diagram against values of ratio Ca:O, categorized in unexposed enamel and the two pooled pH-groups, respectively. The plot visualizes how the enamel is affected by hydrogen peroxide of different pH. The plot of enamel exposed to H2O2 pH<5.5 (square marks) show the most homogenous cluster pattern, considering these parameters. The enamel exposed to H2O2 pH>5.5 (cross marks) has a relatively constant low ratio of C:P, compared to the unexposed enamel, due to carbon lost in oxidation. The pattern of unexposed enamel (circle marks) show a variety of C:P, and a relatively stable ratio of Ca:O.

When studying the chemical composition, a comparison of each element was performed and featured as the differences between unexposed enamel versus exposed enamel in the different pH-groups (Fig. 2). From a summary of these experiments presented in Figure 2, more O is seen to have been lost in exposed enamel at pH>5.5, as opposed to exposed enamel at pH<5.5, and concurrently showed to contain more P and Ca.

The ratios determined from the pooled groups for both the exposed teeth for pH<5.5 and pH>5.5, and ratios from the unexposed enamel. The analysis of the differences of the ratios in unexposed enamel between any of the two pooled groups was performed, and the independent sample t-test determination was used. Since the measured values are semi-quantitative, the ratios of C:O, C:P, Ca:P, Ca:O and Ca:C were calculated. The oxidation process, utilizing hydrogen peroxide, indicates a loss of O and C and a compensatory increase of the elements Ca and P, manifesting itself in differences in ratios. The ratio of Ca:C in exposed enamel was significantly higher, compared to unexposed enamel. This result suggests the compensatory higher Ca content and the loss of carbon from the oxidation process. Loss of carbon is not clearly detectable when element-by-element is investigated ( Table 1).

**Figure 3:**
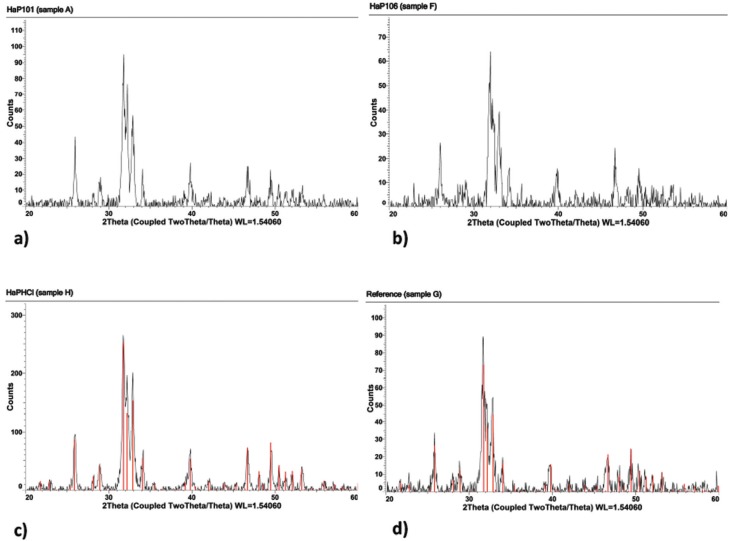
A selection of characteristic XRD diffractograms, based on H2O2 treated and untreated hydroxyapatite samples, is presented. a,b show the diffractograms based on measurements of H2O2 treated samples at pH 4.5 and pH 7.0, respectively. c,d are obtained as reference diffractograms based on powder from untreated samples at pH 3.5 and 7.0, respectively. A least-squares fit of the diffraction pattern from the hydroxyapatite structure is, in addition, applied to the diffractograms from the reference samples and presented in Figures c,d as red peaks.

The analysis of the ratios between the exposed enamel of the two pooled pH-groups showed differences in the ratios of C:P, Ca:P, Ca:O and Ca:C. In detail, the ratio of C:P was seen to be lower for pH>5.5, the enamel for pH>5.5 showed higher ratios of Ca:P, Ca:C, and Ca:O. The change in the chemical content of the enamel surface, when exposed to hydrogen peroxide of various pH-values, is visualized in Figure 3. The most homogenous cluster corresponds to enamel exposed to H2O2 at pH<5.5.

-XRD

A typical XRD diffractogram, obtained from regular human tooth enamel powder at room temperature (denoted sample G), is shown in Fig. 3. By using the Siemens system software “Evaluation®”, the peaks in the diffractogram have been indexed with hkl,, using primarily the hexagonal cell setting. The best fit of four slightly different chemical phases of hydroxylapatite has been calculated and subsequently, the result from the best least-squares fit is also presented in Fig. 3. It is interesting to note that the diffractograms, based on the two samples with the lowest pH (A, B), are best fitted using the monoclinic cell setting. These diffractograms could also be fitted using the hexagonal setting, but the figure of merit was slightly lower. On the other hand, the diffractograms from the two samples with the highest pH-values (E, F) are not only the best fitted with the hexagonal cell setting, but also are better fitted if a fluoride ion is included in the model. The diffractogram from the reference samples denoted G and H, closely resembles the diffractogram from, e.g., samples A and F.

Of particular interest is the impact of the pH-value on the samples, in combination with and without a treatment with hydrogen peroxide. In this study of human enamel using the XRD technique, the parameters 2Ɵ, their corresponding d- values, and subsequently the unit cell parameters, are used as measures of the impact. A preliminary inspection of the results in  Table 2, suggests a continued analysis, in which the samples are divided into two groups; samples with pH-values lower than (or equal to) ca 5.5, and samples with pH-values higher than ca 5.5 units. Basically, the 2Ɵ-values for the reflections increase from a lower level for acidic systems (pH < 5.5) to a higher level for slightly acidic and neutral systems (pH > 5.5).

**Table 2:**
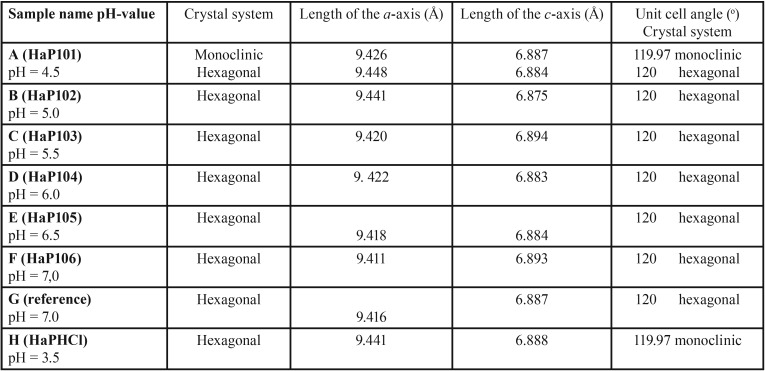
A summary of the results from the least-squares refinement based on the peaks in the different diffractograms.

Since the unit cell volume is an inverse function of sin(Ɵ) according to Bragg’s law, this implies, in a hexagonal crystallographic system, that either the a-axis or the c-axis must decrease. From a least-squares fit of the unit cell parameters to the peaks in the XRD diffractograms (A-F, G-H), it was found that the unit cell parameter a de facto decreases, but the c-axis is essentially constant. For the samples with the two lowest pH units (A, B, H), the average unit cell parameters are a = 9.44 (and b = 18.87 in the monoclinic setting), and c= 6.88 angstrom, respectively, while the same unit cell parameters for the two samples with the highest pH (E, F, G) are a =9.41 and c=6.88 (hexagonal setting).

The null hypothesis is rejected; the oxidation effect seen in enamel differs when exposed to H2O2 pH>5.5, in comparison to enamel exposed to H2O2 pH<5.5.

## Discussion

The aim of this *in vitro* investigation was to show how 3.3% H2O2, at six different pH-values, affects the enamel. When the pH-value is less than 5.5 units, the minerals in enamel are removed by dissolution and the individual crystals in the enamel matrix diminish, resulting in an enlargement of the intercrystalline spaces, which is seen in the SEM and XRD studies.

Hydrogen peroxide is a general oxidizer that reacts with organic matter or biological residual products from the enamel synthesis or bacterial debris in enamel and dentin, although the organic content is low in enamel. Normally, the passage of hydrogen peroxide through enamel is suggested to proceed through the micro pores created by the inter crystalline spaces (15). The hydrogen peroxide will continue to oxidize organic matter in the dentin and continuously into the pulp if H2O2 is still a potent oxidizer 5. In addition to the oxidation effect of H2O2 at different pH-values, the erosive effect of the proton H+, emanating from any weak acid, has to be considered. The critical pH for enamel is frequently stated to be pH 5.5; below this point, demineralization occurs (16,17). Like other *in vitro* studies, this study confirms that the hydroxylapatite is dissolved at pH<5.5 (11,17). The cut of pH at 5.5 set in this investigation, correlates to the critical pH of enamel, theoretically separating demineralized/eroded enamel from unaffected enamel.

-SEM 

The relief of the rods in enamel exposed to H2O2 at pH<5.5 resembles the etched enamel surface previously described (18). This is in accordance with findings when analyzing the effect on the surface of enamel exposed to 10% carbamide peroxide at different pH-values (13).

The loss of minerals cannot be detected in the results of XRMA, where the determination is based on a semi-quantitative measurement, but are still clearly found in the SEM pictures. The interpretation of the chemical content of the surface in exposed enamel at pH<5.5, is that the outermost enamel tissue has been dissolved, and it is the remaining enamel tissue that gives rise to the structural and chemical composition of enamel. The results from XRD suggest a similar difference manifested as a changed signal to noise ratio, discussed in the XRD section. The exposed enamel surface to H2O2 at pH<5.5, experiences both oxidation and erosion, as interpreted from the SEM, XRMA and XRD analyses.

The fidings of oxidation when exposing enamel to H2O2 at pH>5.5 are in agreement with a study showing no difference in the roughness of enamel when exposed to 7.5% H2O2 with pH 6.0 (19). This connects to the loss of carbon, which was found in the analyses using XRMA.

-XRMA 

When enamel is exposed to H2O2 at pH>5.5, an oxidation of the organic or bioorganic matter in enamel occurs. The oxidation contributes to the loss of oxygen, as has been detected using the XRMA analysis tool. The higher values of the Ca and P elements are relative to the actual loss of oxygen and carbon, reflecting the properties of semi-quantitative calculations. No gain of Ca or P is realistic in this study.

The differences in the content of the elements O, P and Ca are seen when the enamel samples are exposed to H2O2 at pH>5.5. This implies that treated enamel with H2O2 at pH>5.5 contributes to an alteration of the element composition in enamel, due to an oxidation.

The ratio of Ca:O in Figure 3 is seen to vary the most in enamel exposed to pH>5.5, since a significant amount of oxygen is lost when the organic matter or the bioorganic moiety in enamel is oxidized. The observed change of the chemical content in enamel, exposed to whitening agents containing hydrogen peroxide, may influence daily clinical work. The oxidation and loss of oxygen in enamel is seen to correspond with the polymerization and reduction of the strength of resin enamel bonds (20). A recommendation of a post-bleaching period of 2-3 weeks before enamel bonding is therefore suggested (20).

-XRD

A general inspection of the XRD diffractograms reveals that the background noise increases when pH is systematically lowered. The loss of the background noise is interpreted as a loss of carbon content due to the fact that the carbon moiety is non-structural and gives rise to an amorphous spread of the X-rays (Figs. 3a-d).

In previous investigations, hydroxylapatite has most frequently been encountered in the enamel structure as hexagonal, with space group symmetry P63/m (international Table No. 176). In older structure investigations, the lattice parameters have been determined in pure hydroxylapatite as a = 9.43 Å, c = 6.88 Å and ƴ = 120o (21,22). Hydroxylapatite also exists in a monoclinic unit cell being slightly more organized. The investigation referred to above (Reyes- Gasca *et al.*) concluded that the human tooth enamel crystals follow the hexagonal cell setting as the phase representative, no matter the stability of the monoclinic hydroxylapatite phase over the hexagonal phase (23). However, the diffractograms, based on the three samples with the lowest pH (A, B, H), are best fitted using the monoclinic cell setting, in contrast to the conclusions in (23). On the other hand, the diffractograms, from the three samples with the highest pH (E, F, G), are best fitted with the hexagonal cell setting.

Of particular interest from the XRD studies is the implication of the unit cell changes along the a-axis, which is elongated when the pH has dropped below ca 5.5 units. The process is driven by the acidity, forcing the enamel surface to erode. At the same time, the amorphous content of CO32- in the structural cavities is protonated, via equilibrium in the water phase, leaves the teeth environment as carbon dioxide.

Due to the *in vivo* complexity, the current investigation is undertaken *in vitro*, with the restriction to show how 3.3% H2O2 at six different pH-values (solutions with pH-values ranging from 4.5 to 7.0 units) affect the enamel topography, and in addition, to show the chemical composition and the crystallinity of human enamel.

## Conclusions

The conclusion is that a determination of the actual pH-value in bleaching treatment, using hydrogen peroxide or carbamide peroxide, is imperative in order to avoid erosion. The recommendation is not to use whitening substances with a pH-value lower than 6.0.
